# Role of Aiolos and Ikaros in the Antitumor and Immunomodulatory Activity of IMiDs in Multiple Myeloma: Better to Lose Than to Find Them

**DOI:** 10.3390/ijms22031103

**Published:** 2021-01-22

**Authors:** Marco Cippitelli, Helena Stabile, Andrea Kosta, Sara Petillo, Angela Gismondi, Angela Santoni, Cinzia Fionda

**Affiliations:** 1 Department of Molecular Medicine, Istituto Pasteur-Fondazione Cenci Bolognetti, Sapienza University of Rome, 00185 Rome, Italy; helena.stabile@uniroma1.it (H.S.); andrea.kosta@uniroma1.it (A.K.); sara.petillo@uniroma1.it (S.P.); angela.gismondi@uniroma1.it (A.G.); angela.santoni@uniroma1.it (A.S.); 2 IRCCS Neuromed–Instituto Neurologico Mediterraneo, 86077 Pozzilli, Italy

**Keywords:** IKZF, Ikaros, Aiolos, IMiDs, Lenalidomide, Multiple Myeloma

## Abstract

The Ikaros zing-finger family transcription factors (IKZF TFs) are important regulators of lymphocyte development and differentiation and are also highly expressed in B cell malignancies, including Multiple Myeloma (MM), where they are required for cancer cell growth and survival. Moreover, IKZF TFs negatively control the functional properties of many immune cells. Thus, the targeting of these proteins has relevant therapeutic implications in cancer. Indeed, accumulating evidence demonstrated that downregulation of Ikaros and Aiolos, two members of the IKZF family, in malignant plasma cells as well as in adaptative and innate lymphocytes, is key for the anti-myeloma activity of Immunomodulatory drugs (IMiDs). This review is focused on IKZF TF-related pathways in MM. In particular, we will address how the depletion of IKZF TFs exerts cytotoxic effects on MM cells, by reducing their survival and proliferation, and concomitantly potentiates the antitumor immune response, thus contributing to therapeutic efficacy of IMiDs, a cornerstone in the treatment of this neoplasia.

## 1. Introduction

The Ikaros zinc-finger (IKZF) protein family consists of five Kruppel-like transcription factors (TFs) (named Ikaros, Helios, Aiolos, Eos and Pegasus or IKZF1-IKZF5) with a well-documented role in lymphocyte development and differentiation [1,2]. Lack of Ikaros proteins from the hemopoietic system leads to an impaired production of B and T lymphocytes and Natural Killer (NK) cells [3]. Consistently, altered function of these proteins has been associated with autoimmunity and cancer [4,5,6]. Indeed, Ikaros proteins act as tumor suppressors in different types of leukemia (where their function is inhibited), whereas they are overexpressed in other malignancies (where they are necessary) to sustain the survival and proliferation of cancer cells, such as in Multiple Myeloma (MM) [7,8].

Multiple Myeloma is an incurable cancer in which malignant plasma cells (PCs) grow and accumulate within bone marrow (BM), causing end-organ dysfunctions and morbidity. MM is a very complex and heterogeneous disease which mainly affects elderly people with a median age of 65 years [9,10,11]. In most cases, MM is preceded by a premalignant condition, named monoclonal gammopathy of undetermined significance (MGUS). The disease conceivably evolves upon accumulation of genetic mutations and deep alterations of the BM microenvironment. Increasing evidence highlights how the compromised functionality of the immune system is crucial for disease progression [12]. A number of mechanisms contribute to prevent anti-myeloma immune response, such as the defective function of dendritic cells (DC) and effector lymphocytes and the expansion of immunosuppressive cells (e.g., myeloid-derived suppressor cells and regulatory T cells, Treg) [13]. Accordingly, some therapeutic approaches to overcome immune dysfunctions have already been approved for MM treatment and several others are currently under investigation. 

A cornerstone in the current MM therapy is represented by immunomodulatory drugs (IMiDs, e.g., thalidomide, lenalidomide and pomalidomide). Lenalidomide is used in the treatment of newly diagnosed patients, in the post-transplant maintenance therapy and in the relapsed/refractory setting, while pomalidomide is used only in the relapsed/refractory disease [14]. IMiDs exert multiple effects on different cellular components present in tumor microenvironment accounting for their profound immunostimulatory properties and direct antitumor activity. Ten years ago, Ito T. and colleagues demonstrated that the cellular target of these drugs is Cereblon (CRBN), a ubiquitous protein which functions as a substrate receptor for the Cullin-4-RING Ubiquitin Ligase (CLR4) complex also including DDB1, ROC1 and CUL4 [15]. Binding of IMiDs to CRBN alters its substrate specificity, thus disrupting or promoting the recruitment and ubiquitination of many proteins [16]. A number of studies described the capability of lenalidomide and pomalidomide to induce CRBN-dependent degradation of Ikaros and Aiolos in different cell types initiating multiple downstream effects, which were shown to be responsible for the antitumor and immunomodulatory properties of these drugs [7,17,18].

Here, we will summarize evidence from the current literature about the complex role of IKZF TFs in regulating the survival and susceptibility of MM cells to immune-mediated attack but also their function in immune effector cells. Moreover, we will discuss how the inhibition of these critical TFs highly contributes to the clinical activity of IMiDs in MM.

## 2. IKZF TFs: Molecular Structure and Transcriptional Activity

All IKZF TFs share the same structural properties. They are characterized by functional domains composed of zinc-finger motifs: an N-terminal DNA-binding domain to bind the consensus sequence A/GGGAA, and two C-terminal dimerization domains to interact with other IKZF proteins. Moreover, shorter IKZF variants can be generated by alternative splicing. These isoforms conserve C-terminal dimerization domains but have a different number of N-terminal zinc fingers. IKZF TFs lacking N-terminal zinc fingers do not bind DNA and function as dominant negative (DN) [19]. 

IKZF proteins can act both as repressors and as activators of gene transcription [20]. Upon binding DNA, they trigger epigenetic regulation and chromatin-remodeling through the direct interaction with two types of remodeling factors, the corepressor (e.g., the nucleosome remodeling and deacetylase complex, NuRD, or C-terminal binding protein, CtBP) and coactivator proteins (Switch/Sucrose-Nonfermentable chromatin-remodeling complex, SWI/SNF). IKZF TFs can repress gene transcription by histone deacetylase (HDAC) dependent and independent mechanisms. They can promote hypo-acetylation of core histones at promoter sites via direct interaction with several corepressor proteins (e.g., NuRD complex ATPase, Mi-2β, Sin3A and Sin3B) which bind HDAC-containing complexes [21]; in addition, they can silence gene expression by recruiting the co-repressor CtBP, which in turn represses transcription initiation through the interactions with the general transcription factors TFIIB and TBP in the transcription pre-initiation complex [22,23]. In a different way, the capability of IKZF TFs to activate gene transcription is due to the formation of activated enhancers and super-enhancers possibly through their interaction with the gene activating complex SWI/SNF [24]. 

IKZF proteins have different expression patterns. Eos and Pegasus are widely distributed in different tissues, while Ikaros, Helios and Aiolos are mainly expressed in immune system cells, and in addition Ikaros and Helios can be found in some non-immune cells, such as erythrocytes. Importantly, several studies demonstrated a role for these proteins in cancer. Impaired functionality of Ikaros, often caused by IKZF DN isoform overexpression, deletions or mutations, has been associated with the development of leukemia (e.g., B and T Acute Lymphoblastic Leukemia, ALL, Acute and Chronic Myeloid Leukemia) [25], indicating a tumor suppressive activity for this transcription factor. In this regard, long-term studies of IMiDs maintenance therapy in MM patients have revealed increased frequencies of hematologic second primary malignancies (SPMs), including B-ALL [26,27], suggesting the involvement of therapy-induced modulation of these TFs. 

In a different way, Ikaros and Aiolos are overexpressed and fully functional in other hematological and solid cancers. For instance, both proteins are highly expressed in MM cells at different stages of disease progression from normal PC, MGUS and MM, independently of genetic subtypes, and are required for the proliferation and survival of these cancer cells [28].

## 3. Aiolos and Ikaros-Dependent Antitumor Activity of IMiDs in MM

**Figure 1:**
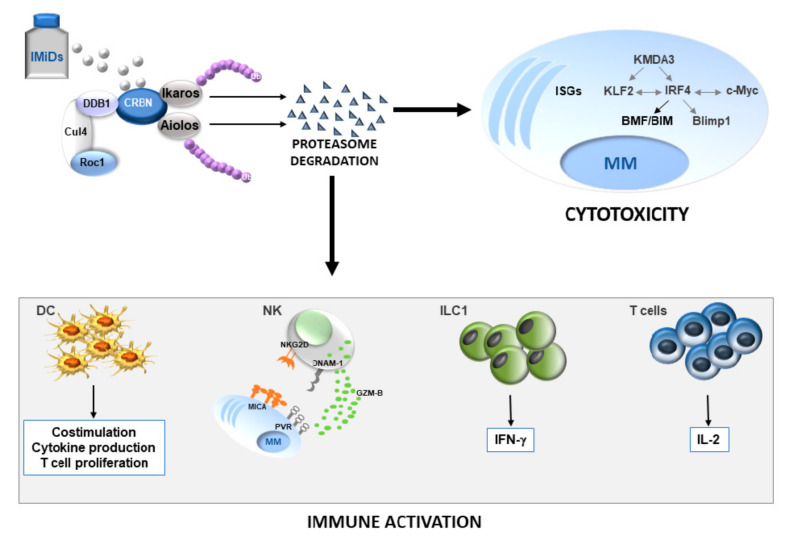
Role of Ikaros/Aiolos loss in anti-MM activity of IMiDs. The drugs induce proteasome degradation of IKZF TFs leading to MM apoptotic cell death and increased antitumor immune response. The grey or black color indicates a decrease or an increase in the expression levels of the depicted molecules, respectively. DC, dendritic cells; NK, natural killer cells; ILC1, innate lymphoid type 1 cells; IMiDs, immunomodulatory drugs; ISGs, IFN-stimulated genes; Ub, ubiquitin.

Immunomodulatory drugs show remarkable antitumor activity in MM through direct inhibition of MM-cell growth in the BM microenvironment and the promotion of immune effector cell function (Figure 1).

The mechanism of action of IMiDs has been shown to rely mainly on the altered substrate specificity of the E3 ubiquitin ligase complex CLR4^CRBN^ and subsequent proteasomal degradation of new substrate proteins. In MM cells more than 240 proteins were identified as CRBN interactors which change their affinity after lenalidomide treatment [29]. In particular, in IMiDs-treated MM cells, there is a significant reduction of the amount of 46 CRBN binding proteins and among them the most downregulated are Ikaros and Aiolos [7,17,30]. IMiDs activate the CRBN-CRL4 E3 ubiquitin ligase to degrade Ikaros and Aiolos. Indeed, these drugs redirect the recruitment of these TFs to CRBN-conjugated E3 ubiquitin ligase leading to their proteolysis via proteasome [16,30]. As part of their transcriptional program, Ikaros and Aiolos serve as anti-apoptotic transcription factors in MM cells and their loss causes reduced proliferation and cell death [7,8]. Indeed, genetic silencing of either Aiolos or Ikaros as well as the overexpression of an Aiolos DN mutant is sufficient to inhibit growth of lenalidomide-sensitive MM cells. Accordingly, low Ikaros expression levels were associated with poor response to IMiDs and shorter overall survival in MM patients treated with these drugs [29].

Although the IKZF TFs target genes responsible for MM cell survival have yet to be fully characterized, different mechanisms have been proposed. 

Importantly, depletion of Ikaros and Aiolos leads to the downregulation of c-Myc and Interferon regulatory factor 4 (IRF4), two known critical TFs/ oncogenes sustaining MM growth and survival [28], and this represents one of the most important mechanisms of the antiproliferative and pro-apoptotic activity mediated by IMiDs in these cells. Notably, in malignant PCs, the sustained expression of c-Myc and IRF4 is a consequence of activating mutations and translocations, and their levels increase during MM progression from early stage MGUS to symptomatic MM, suggesting a major role for these transcription factors in the pathogenesis of the disease [31,32]. A peculiar regulatory circuitry, in which IRF4 and c-Myc regulate each other in a positive feedback loop, sustains the abnormal proliferation of MM cells [33]. Indeed, IRF4 binds to the *c-myc* promoter region promoting its expression, and, in turn, c-Myc transactivates *irf4* gene. Numerous studies have demonstrated that genetic or pharmacologic suppression of either IRF4 or c-Myc expression induces cell cycle arrest and compromises MM viability. c-Myc target genes include regulators of cell cycle (e.g., Cdks, cyclins and E2F TFs), apoptosis and cellular metabolism (e.g., GLUT1) [34]. Importantly, a large group of genes (over 200) involved in the regulation of MM growth and survival are also transcriptionally controlled by IRF4. Among them, a key role is played by Krüppel-like Factor 2 (KLF2) and B-lymphocyte-induced maturation protein-1 (Blimp-1). 

KLF2 is linked to IRF4 by a positive feedback loop involving also a third protein, the histone lysine (K)-specific demethylase 3A (KDMA3) [35]. In this regulatory loop, IRF4 and KLF2 mutually transactivate expression of each other but are also targets of KDMA3, which upregulates both *klf2* and *irf4* gene expression by removing H3K9 methyl marks at their promoters. This protein triad controls key processes of MM pathogenesis, such as survival and homing of malignant PCs to BM. Indeed, as for IRF4, depletion of either KLF2 or KDMA3 triggers apoptosis and reduces the adhesion of MM cells to BM stromal cells, which is critical for sustaining their growth and survival; more intriguingly, the loss of each protein impairs MM cell migratory ability and homing to BM by reducing the expression of the adhesion molecule Integrin alpha-4/beta-7 (ITGB7) [35].

The TF Blimp-1 cooperates with IRF4 in the regulation of PC differentiation and is required for antibody secretion [36]. Due to IRF4 overexpression, MM cells also express high levels of Blimp-1. Compared to PCs, MM cells overexpress the truncated form Blimp-1β which contributes to the deregulation of different genes. Such isoform has a lower repressive activity than wild type protein; consequently, several genes, which are generally inhibited by Blimp-1 in normal PCs, including c-Myc and Bcl6, are expressed in MM cells. IMiDs can regulate Blimp-1 expression and activity through various mechanisms. First, as a direct IRF4 target gene, Blimp-1 expression is inhibited at the transcriptional level. Second, lenalidomide can also promote ubiquitination and proteasomal degradation of Blimp-1 [37]. Third, Aiolos interacts and cooperates with Blimp-1 to repress apoptosis-related genes, such as ASK1, TRAIL, NOXA and KLF10. Indeed, Blimp-1 knockdown leads to MM cell apoptosis as a consequence of the de-repression of the same genes which are upregulated in Aiolos-silenced MM cells [37]. 

More recently, survival dependence of MM cells on IRF4 was attributed to the regulation of two related pro-apoptotic members of the BH3-only subgroup of the BCL2 protein family: BCL2 modifying factor (BMF) and BCL2-like 11 apoptosis facilitator (BIM) [38]. IRF4 directly represses genes encoding BMF and BIM, and, accordingly, the expression of these proteins increases in IRF4-depleted MM cells. More importantly, the absence of BMF alone or in combination with loss of BIM can rescue MM cells from apoptotic cell death upon IRF4 inactivation. 

Although these findings highlight the relevant role for IRF4 in driving the pro-survival and proliferative activity of IKZF TFs in MM cells, a recent study also demonstrated that enforced IRF4 expression failed to rescue cell viability after Ikaros inactivation, thus indicating that multiple mechanisms may concur in causing MM cell death in the absence of IKZF TFs [38]. In this regard, a further contribution may be given by numerous IFN-stimulated genes (ISGs) emerged as additional transcriptional targets of Ikaros proteins in these cancer cells, as revealed by RNA sequencing studies. Indeed, like type I Interferon (IFN), lenalidomide as well as Ikaros inactivation upregulates several ISG in MM cells. IFN-β reduces MM cell viability, but additive cytotoxic effects are observed in combination with either IMiDs or genetic silencing of Ikaros, and correlate with increased expression of interferon stimulated proteins, such as tetratricopeptide repeats (IFIT3) [38]. Moreover, among ISGs, Ikaros and Aiolos repress *CD38* expression through interaction with the nucleosome remodeling and deacetylase complex, and IMiD-induced degradation of these TFs can upregulate CD38 cell surface expression in MM cells, priming them for Daratumumab-induced NK cell-mediated antibody-dependent cellular cytotoxicity (ATCC) [38]. These findings suggest that treatment with IMiDs and IFN may result in synergistic activity in patients with MM and provide an explanation for the improved clinical results that have been reported for the treatment with lenalidomide plus daratumumab [39]. 

In this context, proteasome inhibitors (e.g., bortezomib, carfilzomib) and IMiDs combination therapy have been shown to mediate higher activities in preclinical studies, as well as improved responses in newly diagnosed and relapsed MM [40,41,42,43]. Recent observations indicate that CRBN can be targeted and degraded by SCF^Fbxo7^ E3 ubiquitin ligase [44], and sequential treatment with proteasome inhibitors followed by IMiDs can maintain high levels of CRBN in MM cells thus potentiating Ikaros/Aiolos degradation and cell death [45]. These observations suggest a possible mechanism underlying synergistic preclinical and clinical efficacy of these pharmacological approaches in MM [43,46].

## 4. Aiolos and Ikaros-Dependent Immunomodulatory Activity of IMiDs in MM

Increasing evidence indicates that proteasomal degradation of IKZF TFs plays a critical role in the molecular mechanisms whereby IMiDs reverse MM-mediated immunosuppression (Figure 1). Indeed, these TFs regulate the development and/or function of different immune cells, therefore their depletion can highly impact on anti-tumor immune response.

### 4.1. Role of IKZF Transcription Factors in The Regulation of Innate Immune Response by IMiDs

Key mediators of anti-myeloma immune response include innate and adaptive effector cells, such as the innate lymphoid cells (ILCs) and T lymphocytes. 

Innate lymphoid cells (ILCs) are a heterogeneous immune cell population, lacking antigen specific receptors and myeloid markers, classified in five major groups based on their transcription factors signature and cytokine production that drive their functions [47]. ILCs include Natural Killer (NK) cells, innate lymphoid cell type 1 (ILC1s), type 2 (ILC2s) and type 3 (ILC3s) and lymphoid tissue-inducer cells (LTi). 

NK cells produce large amounts of cytokines and chemokines, and they are also endowed with powerful cytotoxic activity, which depends on the concerted action of numerous inhibitory and activating receptors [48,49]. Several studies provided compelling evidence about the important role for NK cells in the control of MM [50]. Among NK cell activating receptors involved in recognition and killing of malignant PCs, a prominent role is played by the activating receptors natural-killer group 2, member D (NKG2D) and DNAX accessory molecule-1 (DNAM-1) engaged by their ligands on MM cells [51]. Indeed, these cancer cells can express high levels of NKG2D ligands MHC class I chain-related protein A and B (MICA/B) and different UL16 binding proteins (ULBPs), and the DNAM-1 ligands Poliovirus Receptor (PVR) and Nectin-2.

Although the ability of IMiDs to potentiate NK cell function in MM has long been known, the mechanisms responsible for this effect have become to be elucidated recently. Initial observations suggested that IMiDs-induced NK cell activity was due to the capability of these drugs to stimulate IL-2 production by T lymphocytes [52]. Later, Lagrue K. et al. demonstrated that lenalidomide also exerts a direct effect on NK cells. The drug increases IFN-γ production by NK cells isolated from MM patients by augmenting nanoscale rearrangements in cortical actin at the immunological synapse and increasing the proportion of the synapse predicted to be penetrable to IFN-γ vesicles after ligation of the activating receptor CD16 [53]. Importantly, lenalidomide does not trigger IFN-γ production in unstimulated NK cells, thus avoiding inappropriate activation of these effector lymphocytes [53]. More recently, it has been reported that IMiDs-induced potentiation of NK cell activity is predominantly mediated by the increased expression of the cytotoxic molecule granzyme B (GZM-B) through different mechanisms: direct binding and activation of kinase ZAP70 and downregulation of Aiolos. This TF acts as a negative regulator of *gzm-b* gene, therefore pomalidomide-treated NK cells express increased levels of GZM-B due to Aiolos depletion [54]. 

We demonstrated that an additional mechanism accounting for the enhanced NK cell activity after IMiDs treatment consists in the upregulation on MM cells of molecules involved in NK-cell mediated immunosurveillance. Lenalidomide and pomalidomide increase both NKG2D ligand MICA and DNAM-1 ligand PVR membrane expression on human MM cell lines and primary malignant PCs, thus enhancing their susceptibility to NK cell recognition and killing [55]. Silencing of Ikaros and Aiolos is necessary and sufficient for the upregulation of these NK cell activating ligands which occurs at the transcriptional level. These TFs bind to promoter region of *mica* and *pvr* gene and repress their expression. Moreover, MICA expression is negatively regulated also by IRF4. IRF4 shRNA-transduced MM cells expressed higher MICA mRNA and cell surface levels and this correlates with their enhanced capability to stimulate NK cell degranulation in an NKG2D-dependent manner [55]. Thus, IKZF TFs can control the expression of this NKG2D ligand on MM cells via direct and indirect mechanisms.

Relevant immune cell targets of IMiDs in MM include also other ILC subsets [56] and changes in the number and function of ILC populations have been proposed to contribute to MM progression suggesting that they have a role in tumor immunosurveillance. 

Differently from NK cells, other ILC subsets exert mainly an immunomodulatory activity. ILC1 express T-bet and produce IFN-γ in response to IL-12, IL-15 and IL-18. ILC2 differentiation and maintenance is characterized by secretion of IL-4, IL-5, IL-13 and amphiregulin, and depends on Gata3 activation by IL-25 and IL-33. Finally, ILC3 are defined by RORγt expression and have the capacity to produce IL-22 and IL-17 upon stimulation with IL-1β and IL-23. 

A recent study demonstrated that ILCs are among the earliest cell subsets enriched in the tumor microenvironment during the evolution from MGUS to MM even if the signals that lead to enrichment of these cells remain to be clarified [56]. Indeed, an altered ILCs number, subset composition and function were detected in MGUS BM. Compared with healthy donors, MGUS patients in the BM have a higher proportion of ILC1 and a reduction of ILC2 whose percentage increases in circulation. Intriguingly, even though ILCs subset composition remains unaltered during progression to MM, their capability to produce cytokines is significantly compromised, thus suggesting that the functional alteration of these lymphocytes may play an important role in disease evolution. To this regard, IMiDs were shown to boost ILC activity possibly via modulation of Aiolos and Ikaros proteins. Indeed, in vitro and ex vivo studies demonstrated the capacity of pomalidomide to reduce Ikaros and Aiolos levels in ILCs and, more interestingly, to enhance IFN-γ production by ILC1 from both healthy controls and MM patients. These findings indicate that the regulation of ILCs may contribute to immunomodulatory activity of these drugs in MM.

The role of IKZF TFs in the regulation of ILCs functionality has been corroborated in other experimental systems, which had also demonstrated the capability of IMiDs to modulate the activity of these innate lymphocytes [57]. The analysis of IKZF TFs expression in human non-inflamed tonsil and peripheral blood ILC populations showed significantly higher levels of Aiolos in ILC1 and NK cells as compared to ILC2 and ILC3, while Ikaros is expressed by all ILCs subsets and Helios only by ILC3. These observations suggested a differential role of these TFs in the regulation of specific ILC subsets. ILCs are characterized by a high plasticity since they can modify their phenotype and functions in response to local environmental signals [58,59]. In this context, Aiolos and Ikaros emerged as important regulators of ILC3-ILC1/NK cell trans-differentiation. In vitro IL-12 plus IL-1β driven-conversion of ILC3 into ILC1 is accompanied by a specific upregulation of Aiolos, a process inhibited by lenalidomide. The presence of this drug in the trans-differentiation cultures leads to a lower frequency of Aiolos and Ikaros expressing ILCs, downregulates the expression of ILC1 and NK cell related transcripts (e.g., *prf1, gzmb, cd244, lef1, ncr3*) and causes a marked reduction of IFN-γ producing cells. Concomitantly, lenalidomide increases the proportion of IL-22 producing-ILC3 and upregulates several ILC3-related genes (e.g., *rorc, baff, il22 and nrp1*). These findings indicate that IKZF TFs are important for the rheostatic regulation of cytotoxic and immunomodulatory activity of NK/ILC1, but they are also necessary to maintain the functional signature of these subsets. Although our understanding of the role of different ILCs subsets and their plasticity in tumor immunity is only just beginning, these studies suggest that the targeting of Aiolos and Ikaros may be a relevant therapeutic tool to regulate the different ILC activities in cancer. 

Among innate immune cells, DCs have a critical role in antigen presentation for adaptive immune response activation, and a number of studies showed how altered functionality of these cells contributes to MM-immune evasion. MM patient-derived DCs are tolerogenic since they express very low amounts of maturation markers (HLA-DR, CD40, and CD80) and are unable to induce allogeneic T-cell proliferation [60,61,62]. Lenalidomide affects the maturation and functionality of MM patients-derived DCs as antigen presenting cells [63,64]. Moreover, it has been hypothesized that suppression of Aiolos and Ikaros TFs may play an important role in these mechanisms [65]. Upon lenalidomide treatment, DCs differentiated in vitro from BM and PB monocytes of MM patients express higher levels of CD86, HLA-DR and CD209, produce increased amount of cytokines and chemokines (IL-8, TNF, CCL2, CCL5) and have an enhanced ability to stimulate allogeneic T cell proliferation. At molecular level, these stimulatory effects are associated with Aiolos and Ikaros degradation even if the impact of this modulation remains to be clarified. In this regard, a study in a human Ikaros deficiency documented how this TF regulates DC development and function [66]. Individuals harboring a heterozygous mutation of Ikaros have deficiency of plasmacytoid DC (pDC) but the expansion of conventional DC (cDC). These effects may be mediated by Ikaros-dependent regulation of DNA-binding protein inhibitor (ID2) and Basic leucine zipper transcriptional factor ATF-like 3 (BATF3), two TFs driving pDC development and cDC terminal differentiation, respectively. Interestingly, similar effects were also found ex vivo in patients affected by hematological malignancies treated with lenalidomide or during in vitro generation of DCs from primary BM progenitors. As pDCs have been shown to support MM cell growth and mediate myeloma-associated immunodeficiency [67], the negative impact of Ikaros loss or lenalidomide treatment on development of this cell subset may be helpful in antitumor response.

### 4.2. Role of IKZF Transcription Factors in The Regulation of Adaptive Immune Response by IMiDs

Concerning the adaptive immune system, in MM patients the immunostimulatory activity of IMiDs on T cells has been extensively described. IMiDs promote myeloma-specific CD4 and CD8 T cell response and concomitantly inhibit the expansion and the functionality of Treg [68]. Modulation of Ikaros and Aiolos expression by IMiDs could mediate many of these effects since IKZF TFs act as critical regulators of T cell homeostasis. They are required for T cell development and can control T cell activation and differentiation. Initial studies on splenic T cells heterozygous for either an inactivating or a dominant-negative mutation in Ikaros gene demonstrated that this protein can regulate TCR signaling events and control T cell activation. Indeed, Ikaros-mutant T cells are hyperresponsive to TCR and IL-2 signaling leading to a higher proliferative rate compared to wild-type T cells. These findings indicated the capability of this TF to set the threshold for T cell activation [69], thus suggesting that its absence may promote antitumor immune T cell response. Later, a role for Ikaros proteins in cytokine signaling pathways and T cell differentiation has emerged, but conflicting results were reported in different mouse models. Studies using T cells from mice with germline mutations in the Ikaros gene demonstrated that Ikaros negatively regulates T helper 1 (Th1) cell polarization whereas it promotes Th17 and Treg cell differentiation. In this context, Ikaros negatively regulates T-bet, responsible for Th1 polarization, and cytokine production (IFN-γ and IL-2) [70,71,72]. 

Differently, a recent study in an Ikaros conditional knockout (Ikflox) mouse model, in which Ikaros was deleted only in mature T cells [73], showed that the absence of Ikaros impairs only the generation of Treg and results in differentiation towards Th17. Moreover, also in this model, Ikaros deficient-T cells, when activated, express increased levels of pro-inflammatory cytokine genes, including those encoding for IL-2, IFN-γ, TNF-α and GM-CSF, as well as genes associated with type I IFN response. 

These findings confirmed a role for Ikaros as a key regulator of cytokine production by T lymphocytes. More importantly, they suggest that changes in the expression/function of this TF can significantly affect the T cell compartment in MM microenvironment in a way similar to that observed following IMiDs treatment, which is also associated with reduced number and function of Treg and increased conventional T cell activation. Accordingly, it was shown that the loss of IKZFs is responsible for an enhanced IL-2 production by IMiDs-treated T lymphocytes [52]. Ikaros and Aiolos bind the *il2* promoter region and repress gene transcription. Consistently, knockdown of either Ikaros or Aiolos is sufficient to promote *il-2* mRNA expression in T cells and this effect could not be further enhanced by lenalidomide, thus indicating a key role for these IKZF TFs in mediating IL-2 induction by this drug. These observations also suggest that the prolonged/continuous use of IMiDs, in particular as maintenance therapy in MM, could increase the frequency of uncontrolled immunostimulatory activities through induction of chronic inflammatory reactions or autoimmunity [74,75]. 

Notably, these immune stimulating effects of IMiDs persist and also have a therapeutic potential in clinical drug-refractory status. Indeed, in lenalidomide-refractory MM patients, treated with the combination of low dose cyclophosphamide, prednisone and lenalidomide, the degradation of Ikaros and Aiolos in T, B and NK cells was still observed, and it was associated with increased activation of these lymphocytes and better outcome [76]. Similarly, immune activating effects have been described in lenalidomide-refractory MM patients treated with pomalidomide-dexamethasone [77]. These findings highlight the importance of immunomodulatory activity of these drugs and pave the way to new possible combination therapies in which this relevant property of IMiDs can be exploited. 

## 5. Conclusions

Ikaros and Aiolos control multiple aspects of MM biology and anti-tumor immune response. Even though the molecular mechanisms behind this are not fully characterized, the role of these TFs in MM pathogenesis is well documented. Moreover, emerging evidence highlighted how they repress the functional activity of different immune cells, thus restraining immunosurveillance against MM.

To this regard, the identification of these proteins as targets of IMiDs has taken a great step forward regarding MM biology. On one hand, as Ikaros/Aiolos depleting-drugs, IMiDs represent a powerful experimental tool to investigate the complexity of pathways dependent on these proteins in MM as well in immune effector cells. On the other hand, identification of Ikaros and Aiolos gene targets is relevant to better delineate the therapeutic effects of IMiDs as well as to design new pharmacologic agents to achieve better efficacy and overcome IMiDs resistance, one of the major complications in MM therapy. In this context, novel thalidomide analogs, named Cereblon E3 ligse Moldulating Drugs (CELMoDs), have been synthesized and are currently in clinical trials both as monotherapy and in combination with other drugs (NCT01421524; NCT02773030; NCT03374085) [78]. They include avadomide (CC-122), iberdomide (CC-220), CC-885 and CC-92480 [79,80,81,82]. All these drugs bind CRBN with higher affinity than lenalidomide or pomalidomide, thus increasing the amount of CRBN-CRL4 capable to recruit cellular substrates. Consistently, CELMoDs induce a more potent Ikaros and Aiolos degradation in MM cells as compared to lenalidomide or pomalidomide [80,82], and, more importantly, they have strong antitumor and immune stimulatory activities also in relapsed/refractory MM patients, thus representing promising therapeutic opportunities. The development of these new drugs has also revealed that small differences in their chemical structure could significantly affect the substrate ubiquitination, expanding the spectrum of potential druggable targets as well as creating the possibility of selective degradation of proteins in cancer cells. Indeed, although Ikaros and Aiolos are relevant common targets of all these drugs, only lenalidomide induces the degradation of the serine/threonine kinase CK1α [83], while CC-885 also triggers the depletion of the translation termination factor GSPT1 [81]. However, further studies are needed to better identify all substrates of these drugs and define their clinical effects.

## Data Availability

Not applicable.

## Abbreviations

Ikaros family transcription factors (IKZF TFs); Multiple Myeloma (MM); Immunomodulatory drugs (IMiDs); Natural Killer (NK); Plasma cells (PCs); Bone marrow (BM); Second primary malignancies (SPMs); Acute Lymphoblastic Leukemia (ALL); Monoclonal gammopathy of undetermined significance (MGUS); Dendritic cells (DCs); Regulatory T cells (Tregs); Cereblon (CRBN), Cullin-4-RING Ubiquitin Ligase (CLR4); Dominant negative (DN); Nucleosome remodeling and deacetylase complex (NuRD); C-terminal binding protein (CtBP); Switch/Sucrose-Nonfermentable chromatin-remodeling complex (SWI/SNF); Interferon regulatory factor 4 (IRF4); Krüppel-like Factor 2 (KLF2); B-lymphocyte-induced maturation protein-1 (Blimp-1); lysine (K)-specific demethylase 3A (KDMA3); Integrin alpha-4/beta-7 (ITGB7); BCL2 modifying factor (BMF); BCL2-like 11 apoptosis facilitator (BIM); IFN-stimulated genes (ISGs); type I Interferon (IFN); Tetratricopeptide repeats (IFIT3); Innate lymphoid cells (ILCs); Natural-killer group 2, member D (NKG2D); DNAX accessory molecule-1(DNAM-1); MHC class I chain-related protein A and B (MICA/B); UL16 binding proteins (ULBPs); Poliovirus Receptor (PVR); Granzyme B (GZM-B); Plasmacytoid DC (pDC); Conventional DC (cDC); DNA-binding protein inhibitor (ID2); Basic leucine zipper transcriptional factor ATF-like 3 (BATF3); T helper 1 (Th1); T helper 17 (Th17); Ikaros conditional knockout (Ikflox); Histone deacetylase (HDAC); Cereblon E3 ligse Moldulating Drugs (CELMoDs).
